# 1-Ethyl-3-(2,4,6-trimethyl­phen­yl)imidazolium tetra­fluoro­borate

**DOI:** 10.1107/S1600536810027431

**Published:** 2010-07-17

**Authors:** Jin-Tao Guan, Jian-Guo Hou, Zhi-Yong Zhang, Si-Yin Zhao

**Affiliations:** a College of Chemical and Environmental Engineering, Wuhan Polytechnic University, Wuhan 430023, People’s Republic of China; b Department of Biological and Environmental Engineering, Nanchang lnstitute of Technology, Nanchang 330013, People’s Republic of China

## Abstract

The title compound, C_14_H_19_N_2_^+^·BF_4_^−^, was obtained by reaction of 1-ethyl-3-(2,4,6-trimethyl­phen­yl)imidazolium tetra­fluoro­borate with sodium tetra­fluoro­borate. The imidazole ring makes a dihedral angle of 78.92 (13)° with the benzene ring.

## Related literature

For background, reviews and literature related to *N*-heterocyclic carbenes, see: Arduengo *et al.* (1991 ▶); Arduengo (1999 ▶); Wurtz & Glorius (2008 ▶); Haque *et al.* (2010 ▶).
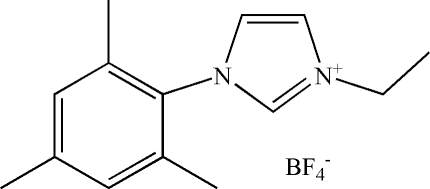


## Experimental

### 

#### Crystal data

C_14_H_19_N_2_^+^·BF_4_^−^
*M* *_r_* = 302.12
Monoclinic, 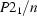

*a* = 7.7637 (7) Å
*b* = 9.1625 (9) Å
*c* = 21.559 (2) Å
β = 91.401 (2)°
*V* = 1533.2 (2) Å^3^
*Z* = 4
Mo *K*α radiation
μ = 0.11 mm^−1^
*T* = 298 K
0.16 × 0.15 × 0.10 mm

#### Data collection

Bruker SMART CCD area-detector diffractometer
Absorption correction: multi-scan (*SADABS*; Sheldrick, 2004 ▶) *T*_min_ = 0.983, *T*_max_ = 0.989
9593 measured reflections
3013 independent reflections
2610 reflections with *I* > 2σ(*I*)
*R*_int_ = 0.026

#### Refinement

*R*[*F* ^2^ > 2σ(*F* ^2^)] = 0.067
*wR*(*F* ^2^) = 0.181
*S* = 1.08
3013 reflections
195 parameters
H-atom parameters constrained
Δρ_max_ = 0.30 e Å^−3^
Δρ_min_ = −0.26 e Å^−3^

### 

Data collection: *SMART* (Bruker, 2001 ▶); cell refinement: *SAINT* (Bruker, 2001 ▶); data reduction: *SAINT*; program(s) used to solve structure: *SHELXS97* (Sheldrick, 2008 ▶); program(s) used to refine structure: *SHELXL97* (Sheldrick, 2008 ▶); molecular graphics: *SHELXTL* (Sheldrick, 2008 ▶); software used to prepare material for publication: *SHELXTL*.

## Supplementary Material

Crystal structure: contains datablocks global, I. DOI: 10.1107/S1600536810027431/jh2177sup1.cif

Structure factors: contains datablocks I. DOI: 10.1107/S1600536810027431/jh2177Isup2.hkl

Additional supplementary materials:  crystallographic information; 3D view; checkCIF report

## Figures and Tables

**Fig. 1.**
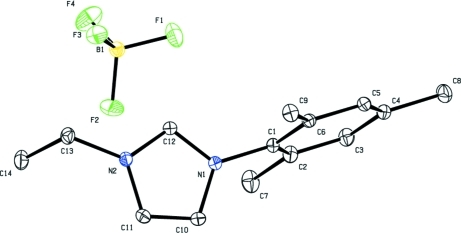
The molecular structure of (I), with atom labels and 50% probability displacement ellipsoids for non-H atoms. H atoms are represented by circles of arbitrary size.

**Fig. 2.**
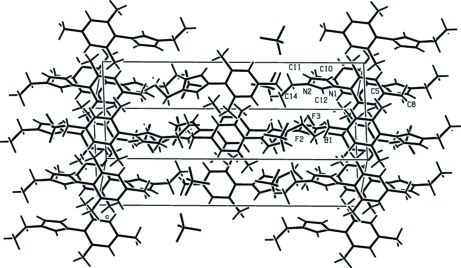
The packing of (I), viewed down the c axis, showing one layer of molecules connected by C—H···F hydrogen bonds (dashed lines).

## supplementary crystallographic information

### Comment

N-Heterocyclic carbenes (NHCs) have been playing an important role as ligands in organometallic chemistry and in catalysis ever since their isolation in the free state by Arduengo and coworkers in 1991 (Arduengo *et al.*,1991 and Arduengo *et al.*, 1999). As part of our research,we designed and synthesized an unsymmetrical carbene precursor imidazolinium salt, namely the title complex (I). The molecular structure of the title complex consists of disubstituted imidazolium cation and tetrafluoroborate anion(Fig. 1). The imidazole ring and benzene ring are oriented at 78.92 (13)°, the imidazole and the plane of the atoms of N2 C13 C14 are oriented at 63.8 (2)°, the imidazole ring slightly deviates from planarity as indicated by the torsion angles: N1—C10—C11—N2 = 1.0 (3)and C11—C10—N1—N2 = -1.0 (3), with a maximum deviation of 0.0056 (18)Å for atom N1.The bond lengths of B—F bonds are ranged from 1.343 (3) to 1.385 (3) Å, and the bond angles of F—B—F are ranged from 108.9 (2) to 111.4 (3)°.

### Experimental

A mixture of 1-ethyl-3-(2,4,6-trimethylphenyl)imidazolium bromide (295.2 mg, 1 mmol) and sodium tetrafluoroborate (142 mg, 1.3 mmol) in THF(10 ml) was stirred for 4 h. The formed precipitate was separated by filtration and washed with Et_2_O and water, dried under vacuum to give an white powder (272 mg). Crystals appropriate for data collection were obtained by slow diffusion of hexane into a solution of the the title compound in dichloromethane at 293 K.

### Refinement

All H atoms were placed in geometrically idealized positions and constrained to ride on their parent atoms with C—H = 0.93 or 0.97 Å; with *U*_iso_(H) = 1.2 or 1.5*U*_eq_(C).

### Crystal data

**Table:**

C_14_H_19_N_2_^+^·BF_4_^−^	*F*(000) = 632
*M**_r_* = 302.12	*D*_x_ = 1.309 Mg m^−^^3^
Monoclinic, *P*2_1_/*n*	Mo *K*α radiation, λ = 0.71073 Å
Hall symbol: -P 2yn	Cell parameters from 3305 reflections
*a* = 7.7637 (7) Å	θ = 2.4–26.5°
*b* = 9.1625 (9) Å	µ = 0.11 mm^−^^1^
*c* = 21.559 (2) Å	*T* = 298 K
β = 91.401 (2)°	Block, colourless
*V* = 1533.2 (2) Å^3^	0.16 × 0.15 × 0.10 mm
*Z* = 4	

### Data collection

**Table:**

Bruker SMART CCD area-detector diffractometer	3013 independent reflections
Radiation source: fine-focus sealed tube	2610 reflections with *I* > 2σ(*I*)
graphite	*R*_int_ = 0.026
phi and ω scans	θ_max_ = 26.0°, θ_min_ = 1.9°
Absorption correction: multi-scan (*SADABS*; Sheldrick, 2004)	*h* = −9→9
*T*_min_ = 0.983, *T*_max_ = 0.989	*k* = −11→9
9593 measured reflections	*l* = −25→26

### Refinement

**Table:**

Refinement on *F*^2^	Secondary atom site location: difference Fourier map
Least-squares matrix: full	Hydrogen site location: inferred from neighbouring sites
*R*[*F*^2^ > 2σ(*F*^2^)] = 0.067	H-atom parameters constrained
*wR*(*F*^2^) = 0.181	*w* = 1/[σ^2^(*F*_o_^2^) + (0.0826*P*)^2^ + 0.7775*P*] where *P* = (*F*_o_^2^ + 2*F*_c_^2^)/3
*S* = 1.08	(Δ/σ)_max_ = 0.026
3013 reflections	Δρ_max_ = 0.30 e Å^−^^3^
195 parameters	Δρ_min_ = −0.26 e Å^−^^3^
0 restraints	Extinction correction: *SHELXS97* (Sheldrick, 2008), Fc^*^=kFc[1+0.001xFc^2^λ^3^/sin(2θ)]^-1/4^
Primary atom site location: structure-invariant direct methods	Extinction coefficient: 0.014 (3)

### Special details

**Table:**

Geometry. All e.s.d.'s (except the e.s.d. in the dihedral angle between two l.s. planes) are estimated using the full covariance matrix. The cell e.s.d.'s are taken into account individually in the estimation of e.s.d.'s in distances, angles and torsion angles; correlations between e.s.d.'s in cell parameters are only used when they are defined by crystal symmetry. An approximate (isotropic) treatment of cell e.s.d.'s is used for estimating e.s.d.'s involving l.s. planes.
Refinement. Refinement of *F*^2^ against ALL reflections. The weighted *R*-factor *wR* and goodness of fit *S* are based on *F*^2^, conventional *R*-factors *R* are based on *F*, with *F* set to zero for negative *F*^2^. The threshold expression of *F*^2^ > σ(*F*^2^) is used only for calculating *R*-factors(gt) *etc*. and is not relevant to the choice of reflections for refinement. *R*-factors based on *F*^2^ are statistically about twice as large as those based on *F*, and *R*- factors based on ALL data will be even larger.

### Fractional atomic coordinates and isotropic or equivalent isotropic displacement parameters (Å^2^)

**Table:**

	*x*	*y*	*z*	*U*_iso_*/*U*_eq_	
B1	0.3268 (4)	0.6442 (3)	0.16502 (13)	0.0525 (7)	
C1	0.7628 (3)	0.8458 (2)	0.04243 (9)	0.0430 (5)	
C2	0.7570 (3)	0.7075 (3)	0.01582 (11)	0.0537 (6)	
C3	0.7342 (3)	0.7003 (3)	−0.04830 (11)	0.0580 (6)	
H3	0.7303	0.6093	−0.0674	0.070*	
C4	0.7172 (3)	0.8245 (3)	−0.08467 (10)	0.0506 (6)	
C5	0.7239 (3)	0.9592 (3)	−0.05564 (10)	0.0459 (5)	
H5	0.7117	1.0429	−0.0797	0.055*	
C6	0.7481 (3)	0.9738 (2)	0.00784 (10)	0.0428 (5)	
C7	0.7746 (5)	0.5706 (3)	0.05432 (14)	0.0803 (9)	
H7A	0.7588	0.4867	0.0281	0.120*	
H7B	0.6890	0.5705	0.0857	0.120*	
H7C	0.8873	0.5674	0.0736	0.120*	
C8	0.6908 (4)	0.8130 (4)	−0.15396 (12)	0.0704 (8)	
H8A	0.5704	0.8002	−0.1637	0.106*	
H8B	0.7540	0.7308	−0.1691	0.106*	
H8C	0.7311	0.9005	−0.1733	0.106*	
C9	0.7608 (3)	1.1231 (3)	0.03680 (12)	0.0576 (6)	
H9A	0.7485	1.1961	0.0051	0.086*	
H9B	0.8709	1.1337	0.0575	0.086*	
H9C	0.6710	1.1346	0.0663	0.086*	
C10	0.9442 (3)	0.8499 (3)	0.14103 (11)	0.0585 (7)	
H10	1.0503	0.8278	0.1243	0.070*	
C11	0.9149 (3)	0.8783 (3)	0.20014 (11)	0.0560 (6)	
H11	0.9968	0.8806	0.2323	0.067*	
C12	0.6688 (3)	0.8903 (2)	0.14954 (10)	0.0432 (5)	
H12	0.5519	0.9012	0.1403	0.052*	
C13	0.6504 (3)	0.9378 (3)	0.26280 (10)	0.0535 (6)	
H13A	0.5305	0.9583	0.2526	0.064*	
H13B	0.7004	1.0248	0.2815	0.064*	
C14	0.6599 (4)	0.8162 (3)	0.30850 (12)	0.0694 (8)	
H14A	0.6102	0.7299	0.2903	0.104*	
H14B	0.5974	0.8425	0.3447	0.104*	
H14C	0.7781	0.7979	0.3200	0.104*	
F1	0.3231 (3)	0.6699 (2)	0.10302 (8)	0.1034 (7)	
F2	0.4792 (3)	0.5802 (3)	0.18106 (10)	0.1111 (8)	
F3	0.3132 (2)	0.7798 (2)	0.19293 (8)	0.0817 (6)	
F4	0.1928 (3)	0.5595 (2)	0.17995 (13)	0.1228 (9)	
N1	0.7890 (2)	0.8590 (2)	0.10897 (8)	0.0433 (4)	
N2	0.7417 (2)	0.9036 (2)	0.20523 (8)	0.0431 (4)	

### Atomic displacement parameters (Å^2^)

**Table:**

	*U*^11^	*U*^22^	*U*^33^	*U*^12^	*U*^13^	*U*^23^
B1	0.0531 (15)	0.0509 (15)	0.0534 (15)	0.0004 (12)	0.0011 (12)	0.0059 (12)
C1	0.0453 (11)	0.0476 (12)	0.0360 (10)	0.0015 (9)	0.0028 (8)	0.0006 (9)
C2	0.0673 (15)	0.0429 (13)	0.0512 (13)	0.0039 (11)	0.0072 (11)	0.0013 (10)
C3	0.0739 (17)	0.0475 (13)	0.0527 (14)	0.0036 (12)	0.0044 (11)	−0.0124 (11)
C4	0.0488 (12)	0.0596 (14)	0.0434 (12)	0.0042 (10)	0.0019 (9)	−0.0033 (10)
C5	0.0456 (12)	0.0490 (13)	0.0430 (11)	0.0031 (9)	0.0014 (9)	0.0061 (9)
C6	0.0402 (11)	0.0445 (12)	0.0437 (11)	0.0005 (9)	0.0018 (8)	−0.0006 (9)
C7	0.124 (3)	0.0468 (15)	0.0703 (19)	0.0093 (16)	0.0099 (17)	0.0075 (13)
C8	0.0803 (19)	0.083 (2)	0.0475 (14)	0.0079 (15)	−0.0039 (12)	−0.0100 (13)
C9	0.0716 (16)	0.0459 (13)	0.0553 (14)	−0.0023 (11)	0.0002 (11)	−0.0014 (11)
C10	0.0392 (12)	0.0832 (19)	0.0530 (14)	0.0065 (11)	0.0012 (10)	0.0082 (12)
C11	0.0452 (12)	0.0752 (17)	0.0474 (13)	0.0004 (11)	−0.0066 (10)	0.0069 (12)
C12	0.0388 (11)	0.0475 (12)	0.0432 (11)	0.0033 (9)	0.0008 (8)	0.0009 (9)
C13	0.0626 (14)	0.0561 (14)	0.0421 (12)	0.0027 (11)	0.0047 (10)	−0.0088 (10)
C14	0.0866 (19)	0.0722 (18)	0.0500 (14)	0.0049 (15)	0.0156 (13)	0.0078 (13)
F1	0.1536 (19)	0.0988 (14)	0.0573 (11)	0.0246 (13)	−0.0050 (11)	0.0061 (9)
F2	0.0887 (14)	0.1184 (17)	0.1254 (18)	0.0440 (12)	−0.0116 (12)	0.0259 (14)
F3	0.0730 (11)	0.0778 (12)	0.0940 (13)	−0.0010 (8)	−0.0021 (9)	−0.0243 (9)
F4	0.1021 (16)	0.0805 (14)	0.188 (3)	−0.0268 (11)	0.0534 (16)	0.0061 (14)
N1	0.0427 (9)	0.0483 (10)	0.0390 (9)	0.0030 (8)	0.0015 (7)	0.0027 (8)
N2	0.0475 (10)	0.0443 (10)	0.0375 (9)	0.0014 (8)	0.0010 (7)	0.0009 (7)

### Geometric parameters (Å, °)

**Table:**

B1—F4	1.343 (3)	C8—H8B	0.9600
B1—F1	1.357 (3)	C8—H8C	0.9600
B1—F2	1.358 (3)	C9—H9A	0.9600
B1—F3	1.385 (3)	C9—H9B	0.9600
C1—C2	1.392 (3)	C9—H9C	0.9600
C1—C6	1.393 (3)	C10—C11	1.326 (3)
C1—N1	1.449 (3)	C10—N1	1.377 (3)
C2—C3	1.391 (3)	C10—H10	0.9300
C2—C7	1.509 (4)	C11—N2	1.371 (3)
C3—C4	1.387 (3)	C11—H11	0.9300
C3—H3	0.9300	C12—N2	1.320 (3)
C4—C5	1.384 (3)	C12—N1	1.326 (3)
C4—C8	1.506 (3)	C12—H12	0.9300
C5—C6	1.383 (3)	C13—N2	1.478 (3)
C5—H5	0.9300	C13—C14	1.488 (4)
C6—C9	1.506 (3)	C13—H13A	0.9700
C7—H7A	0.9600	C13—H13B	0.9700
C7—H7B	0.9600	C14—H14A	0.9600
C7—H7C	0.9600	C14—H14B	0.9600
C8—H8A	0.9600	C14—H14C	0.9600
			
F4—B1—F1	109.8 (2)	H8B—C8—H8C	109.5
F4—B1—F2	111.4 (3)	C6—C9—H9A	109.5
F1—B1—F2	108.9 (2)	C6—C9—H9B	109.5
F4—B1—F3	110.2 (2)	H9A—C9—H9B	109.5
F1—B1—F3	105.8 (2)	C6—C9—H9C	109.5
F2—B1—F3	110.6 (2)	H9A—C9—H9C	109.5
C2—C1—C6	123.0 (2)	H9B—C9—H9C	109.5
C2—C1—N1	119.11 (19)	C11—C10—N1	107.6 (2)
C6—C1—N1	117.87 (19)	C11—C10—H10	126.2
C3—C2—C1	117.1 (2)	N1—C10—H10	126.2
C3—C2—C7	121.0 (2)	C10—C11—N2	107.6 (2)
C1—C2—C7	121.9 (2)	C10—C11—H11	126.2
C4—C3—C2	122.1 (2)	N2—C11—H11	126.2
C4—C3—H3	119.0	N2—C12—N1	109.08 (18)
C2—C3—H3	119.0	N2—C12—H12	125.5
C5—C4—C3	118.3 (2)	N1—C12—H12	125.5
C5—C4—C8	120.9 (2)	N2—C13—C14	112.4 (2)
C3—C4—C8	120.8 (2)	N2—C13—H13A	109.1
C6—C5—C4	122.5 (2)	C14—C13—H13A	109.1
C6—C5—H5	118.8	N2—C13—H13B	109.1
C4—C5—H5	118.8	C14—C13—H13B	109.1
C5—C6—C1	117.1 (2)	H13A—C13—H13B	107.9
C5—C6—C9	120.3 (2)	C13—C14—H14A	109.5
C1—C6—C9	122.62 (19)	C13—C14—H14B	109.5
C2—C7—H7A	109.5	H14A—C14—H14B	109.5
C2—C7—H7B	109.5	C13—C14—H14C	109.5
H7A—C7—H7B	109.5	H14A—C14—H14C	109.5
C2—C7—H7C	109.5	H14B—C14—H14C	109.5
H7A—C7—H7C	109.5	C12—N1—C10	107.65 (18)
H7B—C7—H7C	109.5	C12—N1—C1	125.94 (18)
C4—C8—H8A	109.5	C10—N1—C1	126.31 (18)
C4—C8—H8B	109.5	C12—N2—C11	108.11 (18)
H8A—C8—H8B	109.5	C12—N2—C13	125.41 (19)
C4—C8—H8C	109.5	C11—N2—C13	126.47 (19)
H8A—C8—H8C	109.5		
			
C6—C1—C2—C3	−0.4 (4)	N1—C10—C11—N2	0.6 (3)
N1—C1—C2—C3	−179.0 (2)	N2—C12—N1—C10	1.0 (3)
C6—C1—C2—C7	179.5 (3)	N2—C12—N1—C1	−175.53 (19)
N1—C1—C2—C7	0.9 (4)	C11—C10—N1—C12	−1.0 (3)
C1—C2—C3—C4	−0.3 (4)	C11—C10—N1—C1	175.5 (2)
C7—C2—C3—C4	179.8 (3)	C2—C1—N1—C12	−103.7 (3)
C2—C3—C4—C5	0.2 (4)	C6—C1—N1—C12	77.7 (3)
C2—C3—C4—C8	−179.3 (2)	C2—C1—N1—C10	80.4 (3)
C3—C4—C5—C6	0.5 (3)	C6—C1—N1—C10	−98.3 (3)
C8—C4—C5—C6	−179.9 (2)	N1—C12—N2—C11	−0.7 (3)
C4—C5—C6—C1	−1.1 (3)	N1—C12—N2—C13	−179.9 (2)
C4—C5—C6—C9	177.8 (2)	C10—C11—N2—C12	0.0 (3)
C2—C1—C6—C5	1.1 (3)	C10—C11—N2—C13	179.2 (2)
N1—C1—C6—C5	179.73 (18)	C14—C13—N2—C12	115.6 (3)
C2—C1—C6—C9	−177.8 (2)	C14—C13—N2—C11	−63.4 (3)
N1—C1—C6—C9	0.8 (3)		
